# Recent Progress in the Application of Metal Organic Frameworks in Surface-Enhanced Raman Scattering Detection

**DOI:** 10.3390/bios13040479

**Published:** 2023-04-16

**Authors:** Haojia Qin, Shuai Zhao, Huaping Gong, Zhi Yu, Qiang Chen, Pei Liang, De Zhang

**Affiliations:** 1 College of Optical and Electronic Technology, China Jiliang University, Hangzhou 310018, China; 2 National Key Laboratory for Germplasm Innovation and Utilization for Fruit and Vegetable Horticultural Crops, Key Laboratory of Horticultural Plant Biology, Ministry of Education, College of Horticulture & Forestry Sciences, Huazhong Agricultural University, Wuhan 430070, China; 3 College of Metrology and Measurement Engineering, China Jiliang University, Hangzhou 310018, China

**Keywords:** MOF, SERS, synthesis, environmental detection, biomedical science

## Abstract

Metal–organic framework (MOF) compounds are centered on metal ions or metal ion clusters, forming lattices with a highly ordered periodic porous network structure by connecting organic ligands. As MOFs have the advantages of high porosity, large specific surface area, controllable pore size, etc., they are widely used in gas storage, catalysis, adsorption, separation and other fields. SERS substrate based on MOFs can not only improve the sensitivity of SERS analysis but also solve the problem of easy aggregation of substrate nanoparticles. By combining MOFs with SERS, SERS performance is further improved, and tremendous research progress has been made in recent years. In this review, three methods of preparing MOF-based SERS substrates are introduced, and the latest applications of MOF-based SERS substrates in biosensors, the environment, gases and medical treatments are discussed. Finally, the current status and prospects of MOF-based SERS analysis are summarized.

## 1. Introduction

Surface-enhanced Raman spectroscopy (SERS) is a kind of molecular fingerprint spectrum in which the frequency variation is related to the unique molecular vibration information, and the peak intensity is proportional to the molecular concentration [1]. The earliest discovery of SERS was in 1974 when Fleischmann and his team found the adsorbed pyridine molecules on the rough silver electrode surface can produce the Raman scattering phenomenon with good effect. However, they believe that the enhancement of the Raman signal is due to the increase in the specific surface area of the metal electrode [2]. Later, in 1977, Jeanmarie and Albrecht also independently observed this phenomenon. They calculated experimentally that the Raman signal enhancement was about five to six orders of magnitude and believed that the Raman signal could not be increased by a large specific surface area alone. They proposed that this phenomenon was caused by the resonance Raman effect excited by plasma, that is, the surface-enhanced Raman scattering (SERS) effect [3,4]. Thus, SERS can be used for qualitative and quantitative analysis. Benefiting from the features of high sensitivity, accuracy and speed [5], SERS has been widely employed for the detection of harmful substances [6], environment analysis [7], artwork appraisal [8], pharmaceutical analysis, biomolecular identification [9] and so on [10].

Up to date, the electromagnetic enhancement mechanism (EM) and chemical enhancement mechanism (CM) of SERS are most accepted by researchers [11,12]. The electromagnetic enhancement is due to local surface plasma resonance (LSPR), which is provided by the coupling of the metal surface and free photons in space, allowing Raman signal improvement of 10^4^–10^14^ or higher fold [13]. Traditional precious metal materials (Au, Ag, Cu, etc.) are the typical representation of electromagnetic field enhancement. For the metallic nanostructure, the quasi-free electroclouds are pulled back and forth harmonically with the incident EM field against the Coulombic force between the electron and the nuclei, leading to an enhancement of the localized EM field. The plasmonic nanostructures with sharp tips, narrow gaps and aggregates generally provide more enhanced Raman scattering, and their localized surface plasmon resonance (LSPR) wavelength can be tuned by the factors, e.g., NP composition, size, alloy proportion, etc. [14]. The chemical enhancement from SERS is caused by the charge transfer between the metal substrate and the target absorbed on the surface of the metal substrate [15]. Most CM processes involve the following three points: (i) the increased polarizability of the molecules involved in the process, (ii) molecular electronic excitation promoted by the photon excitation, or (iii) a special condition in which the orbitals involved in the electronic excitation are localized in different parts of the system (corresponding to charge transfer transitions), i.e., the electron initially localized in a metal orbital is promoted into a molecular orbital localized on the organic molecule [14]. In some specific systems, CM can make a significant contribution to the SERS effect [16]. The substrate plays an important role in the SERS effect, whether there is electromagnetic or chemical enhancement. Consequently, a considerable number of researchers explored and constructed effective SERS substrates to meet the requirement of the application. For example, Wu et al. [17] use improved seed growth methods to prepare silica nanoparticles and further, synthesize silica/silver nanoparticle composites (SANC). Strawberry-like SANC structure provides rich SERS “hot spots”, and the detection capacity of acrylamide, a starch frying product, can reach 10–11 M. In terms of the environment, Gao et al. [18] combined the advantages of cotton fabric and precious metal materials and coated the cotton fabric with a silver nano layer by magnetron sputtering. The substrate’s SERS detection capability for thiram in real samples can reach 10^−7^ M, which is lower than the national standard of 10^−5^ M.

Through a great deal of effort, precious metal substrates with various morphologies or chemically and physically modified have been successfully applied to ultra-trace detection [5]. However, when facing the real application, these noble substrates reveal the bottlenecks of poor stability, high cost, matrix interference and poor affinity between noble metal surfaces and analytes, which hinder the further development and application of SERS. Accordingly, SERS substrates have been developed from single-component noble metals to composite substrates of various materials in the past few decades [19,20]. Integrating with new materials to construct SERS substrates can overcome the above problems in practical testing and be effective in improving the SERS performance. Compared with the existing materials, composite materials formed by integrating with noble metals have tremendous inherent advantages in realizing highly effective SERS enhancement because composite materials may have the ability of selective capture as well as rapid absorption and enrichment [21,22]. SERS composite substrates based on metal–organic frameworks (MOFs) have been demonstrated to be an effective alternative to realize the above advantages in SERS application.

The metal–organic framework is a kind of porous coordination polymer built from organic linkers and metal ions or clusters via coordination bonds [23]. Usually, MOFs are highly ordered periodic networks, which allow them to possess large specific surface areas, more active metal sites, superior adsorption and excellent stability [24,25]. Therefore, the unique physical and chemical properties of MOFs have effectively expanded their application in adsorption [26], gas storage and separation [27], drug delivery and catalysis [28,29,30,31]. Apart from these traditional areas, MOFs-based materials for SERS sensing have attracted great enthusiasm from researchers and achieved remarkable development [32,33,34]. For example, the combination of noble metal and MOF materials not only separates target and impurities to reduce monitoring interference but also obtains effective hotspot area due to the excellent molecular adsorption and aggregation [35]. More specifically, some MOF materials themselves can be used as an enhanced substrate for SERS application, which can greatly reduce the cost of substrate [36]. MOF-based substrate for constructing SERS platforms plays an important role with multiple effects, including target concentration enrichment, selective capture, chemical enhancement as well as synergistic enhancement. Firstly, in terms of screening, the MOF–SERS substrate has a large surface area that enables molecular concentration, and the uniform cavity has a molecular sieve effect, making the substrate size selective [37]. Secondly, in terms of enrichment, MOF-based SERS-active substrates can absorb/enrich target molecules and not be disturbed by humidity so that the platform can easily deal with the complex background interference from the human body’s exhales fluids and tissues [38]. Finally, in terms of sensitivity, MOFs have a unique structure, good chemical stability and thermal stability. SERS analysis based on MOFs shows higher sensitivity, stability and reproducibility. All these above multiple effects can contribute to the change of speed or intensity, specificity and the interaction mode of analyte in the SERS detection system, which can stimulate more scientific problems and expand SERS application. Recently, Zhao et al. [39] synthesized gold nanorods/organometallic frameworks for photo-enhanced peroxidase-like catalysis and surface-enhanced Raman spectroscopy (SERS). Under local surface plasmon resonance (LSPR), the enzyme-like activity of AuNRs/Fe MOF hybrid was significantly enhanced because the hot electrons generated on the surface of AuNRs were transferred to Fe MOF, and the Fenton reaction was activated through Fe^3+^/Fe^2+^ conversion, and the recombination of hot electrons and holes was prevented. AuNRs/Fe MOF causes chemical and electromagnetic enhancement of the Raman signal, and the detection limit of MB concentration is 9.3 × 10^−12^ M.

Recently, several reviews have summarized the role of MOF in SERS and the synthesis methods of MOF [25,40], but none have detailed and elaborated the synthesis strategies of MOF-based substrate from SERS enhancement and its SERS application. Therefore, the review primarily concentrated on summarizing the varieties of MOF-based SERS substrates based on the analysis of synthesis strategies from SERS enhancement and the representative applications of MOF–SERS platforms. In the end, the challenges and future development trends of MOF–SERS platforms are featured.

## 2. Varieties of MOF-Based SERS Substrate

Generally, traditional SERS substrates can be divided into two basic substrate categories: the noble metal substrate and the semiconductor substrate. Metal substrates, especially precious metals, such as Au, Ag, and Cu, can bring strong plasma coupling, which leads to SERS signal enhancement, mainly due to the presence of EM. In contrast, due to the presence of CM, some semiconductor SERS substrates such as W_18_O_49_, Cu_2_O, and MoS_2_ [41,42,43,44] have also shown to exhibit significant Raman enhancement, in which charge transfer at the semiconductor analyte interface plays a major role. Different from these typical SERS substrates, MOFs may be a very interesting material in SERS, which is worthy of in-depth exploration according to their unique characteristics. We mainly explore the following three aspects.

### 2.1. MOF Combined with Precious Metals as SERS Substrate

**Figure 1:**
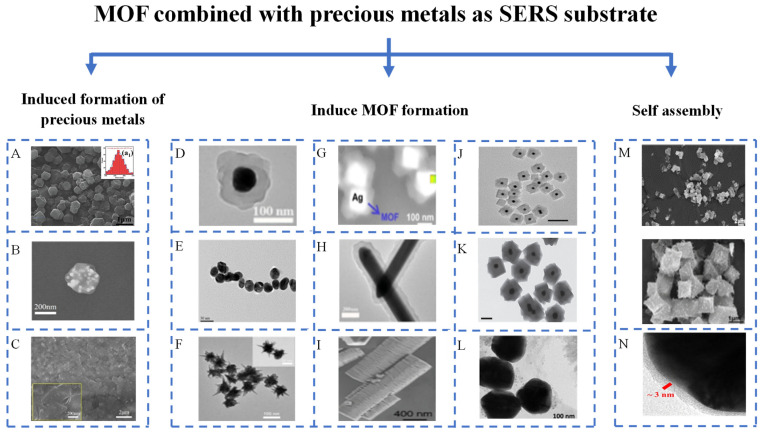
(**A**–**C**) SERS substrate synthesized by inducing precious metal nanoparticles to fabricate in the MOF internal and external surfaces [45,46,47]. (**D**–**F**) SERS substrate with gold nanoparticles as core and MOF as shell [48,49,50]. (**G**–**I**) SERS substrate with silver nanoparticles as core and MOF as shell [51,52,53]. (**J**–**L**) SERS substrate with gold/silver composite nanoparticles as the core and MOF as the shell [54,55,56]. (**M**,**N**) Self assembled SERS Substrates of MOF and Noble Metal Nanoparticles [57,58].

Currently, MOF–SERS substrates are predominantly composed of a combination of MOF and precious metal nanoparticles. The combination of MOF and metal substrate creates a synergistic effect that significantly enhances the performance of SERS analysis. To overcome the challenges faced in SERS analysis, it is crucial to design and synthesize MOF–SERS substrates with exceptional SERS activity. This approach offers an effective solution to improve the accuracy and sensitivity of SERS analysis. This article outlines the framework for using Metal–Organic Frameworks (MOFs) in combination with precious metals as Surface-Enhanced Raman Scattering (SERS) substrates, as shown in Figure 1. This framework consists of three main components. First, the synthesis of noble metal nanoparticles induced by the internal and external surfaces of MOF is an efficient method. The size, shape and distribution of nanoparticles can be adjusted by controlling the chemical properties and structure of MOF surface. Secondly, MOF growth can be induced around noble metal nanoparticles to achieve directional growth and control the position of nanoparticles in MOFs, resulting in control over material properties. Finally, the self-assembly of MOFs and noble metal nanoparticles can lead to the orderly assembly of the two materials, resulting in new nanomaterials with superior performance and promising applications.

#### 2.1.1. Synthesis of Precious Metal Nanoparticles Induced by the Internal and External Surfaces of MOF

MOF was first synthesized, then dispersed in the precursor of noble metal nanoparticles. Because of the large specific surface area of MOF, metal precursors can be induced to form metal nanoparticles and then be fixed on the surface in large quantities, forming a large number of SERS active sites. Moreover, the size of metal nanoparticles can be changed by adjusting the content of metal precursor. Combined with the adsorption capacity of MOF, the SERS detection capacity can be effectively improved.

First, metal nanoparticles were synthesized in situ on the outer surface. Jing et al. [33] synthesized silver nanoparticles (AgNPs) in situ on the surface of a metal–organic skeleton MIL-101 (Fe) for the first time. The SERS substrate prepared combines many Raman hotspots between high-density AgNPs and the excellent adsorption performance of MOF, which can effectively concentrate the analyte in the Raman hotspots between adjacent Ag NPs, making it an excellent SERS substrate for high-sensitivity detection. Su et al. [45] prepared a 3D ZnO/Ag substrate using ZnO nanoparticles (NPs) derived from porous zeolite imidazole skeleton (ZIF-8) and then evaporated to induce the self-assembly of AgNPs on it. This substrate can detect a variety of environmental pollutants through simple and effective methods (Figure 1A). R6G as a probe molecule, the detection limit can reach 10^−13^ M. Secondly, metal nanoparticles are embedded on the inner surface, and solution impregnation is a more common method. Shao et al. [46] encapsulated AuNP into the inner surface of Fe_3_O_4_/MIL-101 (Cr) by solution immersion method. They first dropped the Fe^3+^/Fe^2+^ mixture, injected precipitant NH_3_·H_2_O when boiling, and then separated and dried it with a magnet to obtain a magnetic Fe_3_O_4_/MIL-101 (Cr) composite. Then it was suspended in the aqueous solution of chloroauric acid (HAuCl_4_) to ensure the solution penetration of noble metal nanoparticles before adding sodium citrate solution to reduce the precursor. The optimum Au/Fe_3_O_4_/MIL-101 (Cr) (AF-MIL) SERS substrate was obtained by adjusting the concentration of chloroauric acid and the reduction time (Figure 1B). In addition to using sodium citrate, sodium borohydride and other reducing agents can also be used to obtain metal nanoparticles through external light sources, such as ultraviolet lamps. For example, Shao et al. [47] have also synthesized Ag/MIL-101 (Cr) thin film material SERS substrate (Figure 1C) by secondary growth method and reduction reaction. The MIL-101 (Cr) film was grown on foil for the second time, and the MIL-101 (Cr) film immersed in silver nitrate aqueous solution was irradiated with an ultraviolet lamp. The silver ions were reduced to silver nanoparticles and attached to the surface of the MIL-101 (Cr) film. Through Raman detection of probe molecule 4-ATP, it was found that it could still be detected at the concentration of 10^−11^ M.

#### 2.1.2. Induced MOF Growth around Precious Metal Nanoparticles

First, noble metal nanoparticles are synthesized, and then MOF is induced around noble metal nanoparticles. When noble metal nanoparticles are combined with MOF precursors, the catalytic active sites on the surface of the noble metal nanoparticles can facilitate the polymerization of the MOF precursors, ultimately accelerating the formation of the MOF structure. Additionally, the unique morphological characteristics of the noble metal nanoparticles can serve as a template to guide the arrangement and assembly of MOF precursors, leading to the formation of a highly ordered MOF structure. These mechanisms work in tandem to produce highly controllable MOF structures. To achieve optimal control over this process, factors, such as the shape and size of the noble metal nanoparticles, the concentration and reaction conditions of the MOF precursors and surface modifications of the noble metal nanoparticles, can all be precisely manipulated. In such composite structures, the core–shell substrate is a common one. The MOF structure with a noble metal core can effectively avoid the problem of the aggregation of noble metal nanoparticles in the SERS substrate, and the thickness of the MOF shell can be effectively controlled. Combined with the diversity of MOF materials on the outer surface and the enhancement effect of noble metals in the core, the stability and sensitivity of the SERS substrate can be greatly improved.

In traditional SERS analysis, gold as the substrate can produce more stable and reproducible spectra, so there are many methods to make gold nanoparticles as the core of SERS substrate in MOF structures. For example, Chen et al. [48] added the precursor of ZIF to AuNPs solution to form a core–shell structure Au@ZIF-8 (Figure 1D), and the thickness of the MOF shell can be precisely adjusted from 3–50 nm. As an attractive core–shell structure, it can monitor the change of the toluene SERS spectrum with time. Cai et al. [49] successfully prepared AuNPs@IP6 @MIL-101 (Fe), using the layer-by-layer assembly method (Figure 1E), increased the number of “hot spots” and the direct detection of urotropine can reach 5 × 10^−10^ M. Fu et al. [50] in situ grew a porous zeolite imidazole skeleton-8 (ZIF-8) shell (Figure 1F) on the gold nanostars (AuNSs) for capturing and changing the route of formaldehyde molecules. Compared with the current SERS detection methods, this synthetic core–shell with special aperture AuNS@ZIF-8 nanocomposites can enable formaldehyde molecules to reach the metal surface through the ZIF-8 channel. With the help of ZIF-8 housing, AuNS@ZIF-8 shows extremely high sensitivity to formaldehyde molecules, and the lowest detection level is almost one part per billion (ppb).

Compared with gold nanoparticles, the MOF core–shell structure with a silver core has higher accuracy. Phan Quang et al. [51] used the silver nanocube as the building block of plasmon excimer nanoparticles and then encapsulated the silver nanocube (Figure 1G) with zeolite imidazolium ester skeleton material 8 (ZIF-8), which absorbed molecules, to synthesize a three-dimensional plasma(Ag@MOF)SERS hot spots with micron thickness that can actively absorb and rapidly detect aerosols, gases and volatile organic compounds. Liu et al. [52] synthesized the Ag@ZIF-8 nanowires. After the Ag nanowires are coated with PVP, they are directly mixed with the precursor of ZIF-8 in methanol to generate a ZIF-8 shell on the surface of the Ag nanowires (Figure 1H). Combined with the excellent adsorption capacity of ZIF-8 shell to butanol and the unique plasma photothermal effect of the Ag nanowire core, the core–shell structure Ag@ZIF-8 successfully realized the renewable adsorption and separation of low concentration butanol in aqueous solution under solar irradiation. Yang et al. [53] successfully developed a new multifunctional stacked hexagonal prism Ag@Ni-MOF-1 as an integrated SERS platform (Figure 1I). Based on the biomimetic interaction between DA(dopamine) quinone and cysteine Ag@Ni-MOF-1, the SERS of cysteine in mouse brain microdialysate is highly selective and sensitive.

Finally, it is a composite structure with gold and silver nanoparticles as the core in the core and shell structure of MOF. For example, Yang et al. [56] made a new array-assisted SERS microfluidic chip, which is composed of MOF material (zeolite imidazole skeleton 8 (ZIF-8)) and Au@Ag. The nanocube is composed of cysteamine (CA) as a gas-trapping agent (Figure 1J). Due to the existence of the array, the gas molecules are first guided to the vicinity of the sensing interface, then decelerated by the porous material ZIF-8, and finally captured by the recognized molecules. Compared with traditional detection methods, the detection limit can be reduced by two orders of magnitude. Zheng et al. [54] encapsulated gold nanorods Au@Ag SERS substrates with the core–shell structure was prepared by core–shell nanorods, gold nanostars and gold nanospheres. Lai et al. [55] prepared core–shell Au@Ag Nanoparticles (Ni-MOF-Au@AgNPs) (Figure 1L). The two-dimensional MOF nano sheet enables the substrate to rapidly adsorb and separate positively charged targets, so it can achieve rapid adsorption, separation and selective quantification of thiram, diquat and paraquat. The entire analysis process only takes 30–45 min.

#### 2.1.3. Self-Assembly of MOF and Noble Metal Nanoparticles

There are two kinds of self-assembly methods. The first is to synthesize MOF and metal nanoparticles, respectively, and then to form composite materials through self-assembly; The second type is to directly mix the precursor of nanoparticles and the precursor of MOF to form composite materials. The self-assembly method is simple and effective. There are many systematic methods to synthesize MOF and metal nanoparticles, so the size and content of MOF and metal nanoparticles can be effectively controlled in the self-assembly process. This method has great application prospects in sensors.

First, it is the first method to synthesize MOF and nanoparticles, respectively. For example, Zheng et al. [57] synthesized MIL-101 (Fe) by hydrothermal reaction and then synthesized AgNPs by reduction. The composite substrate AgNPs/MIL-101 (Fe) was obtained by self assembly of AgNPs onto the MIL-101 (Fe) surface (Figure 1M). The volume ratio of MIL-101 (Fe) to AgNPs was adjusted to 1:2 to obtain the best substrate. The substrate can detect paraquat up to 10^−12^ M. The second method is direct synthesis. Zhang et al. [58] directly mixed gold with precursors of MOF-74, DMF, PVP and ethanol to synthesize core–shell at 120 °C AuNP@MOF-74 Composite materials (Figure 1N). In the self-assembly process, HAuCl_4_ is first reduced by DMF to form Au nanoparticles, and then MOF-5 starts to grow on the surface of PVP-coated Au nanoparticles to form a AuNP@MOF-5 core–shell composite. The prepared composite materials have good SERS activity and can detect very low concentrations of nitrophenyl-thiophenol, which broadens the research field of composite nanoparticles designed for sensitive detection of surface-enhanced Raman spectroscopy.

### 2.2. MOF as SERS Substrate Alone

**Figure 2:**
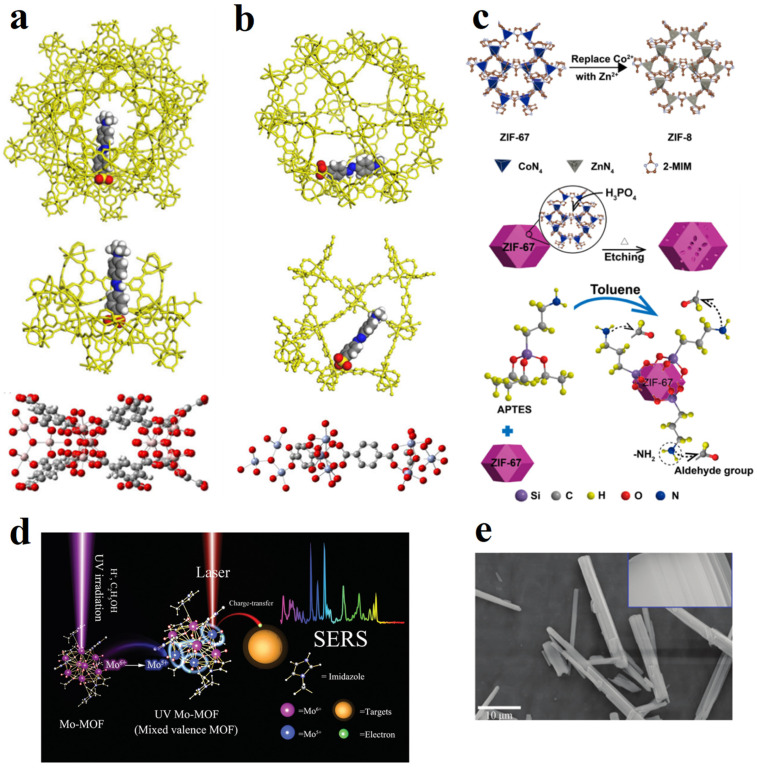
Single MOF as SERS substrate and semiconductor combined with MOF as SERS substrate (**a**) The molecular adsorption orientation of methyl orange in the MO+MIL-100 system is almost perpendicular to the bottom pentagonal ring [36]; (**b**) The molecular adsorption orientation of methyl orange in the MO+MIL-101 system is close to the bottom pentagonal ring [36]; (**c**) Metal ion replacement, optimization of phosphoric acid etching pore structure and surface modification [32]; (**d**) Synthesis of ultraviolet Mo-MOF and its SERS effect [59]; (**e**) SEM image of Mo-MOF [59].

Generally, most MOFs do not have SERS activity without the synergistic effect of noble metal nanoparticles. However, Yu et al.’s research on single MOF provides powerful help for the CM enhancement of SERS. Yu et al. conducted a study on the SERS signals observed on two types of mesoporous MOF substrates, MIL-100 (Al) and MIL-101 (Cr), using methyl orange as a probe molecule. The researchers were able to achieve SERS enhancement without the addition of metal sols or enhancers, with an EF value of approximately 60 for MIL-100 (Al) and an estimated EF value of 120 for MIL-101 (Cr). The SERS effect was attributed to the charge transfer effect between metal clusters and methyl orange molecules in MOFs. The researchers also explained the SERS effect of mono component MOFs (MIL-100 and MIL-101) using spectral distribution and DFT calculations in Figure 2a,b. In addition, they also removed organic bonds in MOFs through high-temperature and O_2_ plasma treatment, proving that MOF derivative metal oxides can also be used as active substrates for generating SERS signals. This result indicates that metal oxide clusters can act as SERS active sites, and the SERS effect observed on MIL-100 (Al), MIL-100 (Cr), and MIL-101 (Cr) on methyl orange can all be attributed to the charge transfer effect between metal clusters and methyl orange molecules in MOFs. From the test results, it can be seen that using pure MOF as a SERS substrate directly cannot reach the excellent detection limit. However, MOF materials can be transformed from non-SERS active to high SERS active substrates by adjusting the metal center, organic linker and skeleton topology. Sun et al. [32] optimized the substrate by metal ion replacement, pore structure optimization and surface modification of ZIF-67. The results showed that the morphology of acid-treated ZIF-67 rapidly changed from solid state to hollow structure, and the structure changed from microporous to mesoporous. The widening of pore size distribution can reduce the blockage degree of adsorbents to pores and reduce the impact on the adsorption of trace adsorbents. With R6G as the probe molecule, the detection limit can reach 10^−8^ M (Figure 2c). This selective SERS enhancement phenomenon is caused by molecular resonance, inter-band transition resonance, photo-induced charge transfer transition and ground state charge transfer. In addition to changing the internal structure of MOF, SERS activity can also be excited by external light sources. In the recent two years, Chen et al. [59] developed a mixed valence molybdenum-based metal organic framework (Mo-MOF) without any embedded noble metal nanoparticles or other enhancement cofactors as SERS substrates (Figure 2d). The substrate (Figure 2e) is irradiated by ultraviolet light to form mixed valence Mo-O clusters. Oxygen vacancies in Mo-MOF can induce the formation of SERS active sites. The porous structure and large specific surface area of Mo-MOF endows it with the ability to adsorb and anchor SERS molecular probes, further improving the SERS signal.

### 2.3. MOF Combined with Other Semiconductor Materials as SERS Substrate

In addition to precious metals and MOF composite as SERS substrates, researchers also try to use semiconductor materials, such as GO, SiO_2_ and MOF, to combine. Liu et al. [60] used the one-pot method to prepare the composite graphene oxide (GO)/MIL-101 (Fe). The surface area of GO/MIL-101 (Fe) decreases due to the blockage of some GO in the composite, but its active sites increase. Agglomerated GO can prevent organic ligands from coordinating with Fe^3+^; thus, more positive charges appear on the surface of the composite. Therefore, the maximum adsorption capacity of GO/MIL-101 (Fe) on methyl orange (MO) is better than MIL-101 (Fe).

Overall, MOF based SERS substrates are more stable and have higher sensitivity. Therefore, Table 1 summarizes the research progress of different types of MOF–SERS substrates in recent years.

## 3. Application of MOF-SERS Platform

### 3.1. Environmental Detection

With the rapid development of industry in recent decades, environmental pollution has become a hot issue that people need to solve presently [63]. The concentration of most harmful substances in the environment is low and challenging to detect [64,65]. Due to the customizable and functional structure of MOFs, a variety of interactions can be customized for the selective enrichment and detection of trace analytes [66,67]. The SERS substrate based on MOFs has successfully detected organic pollutants in the water environment.

**Figure 3:**
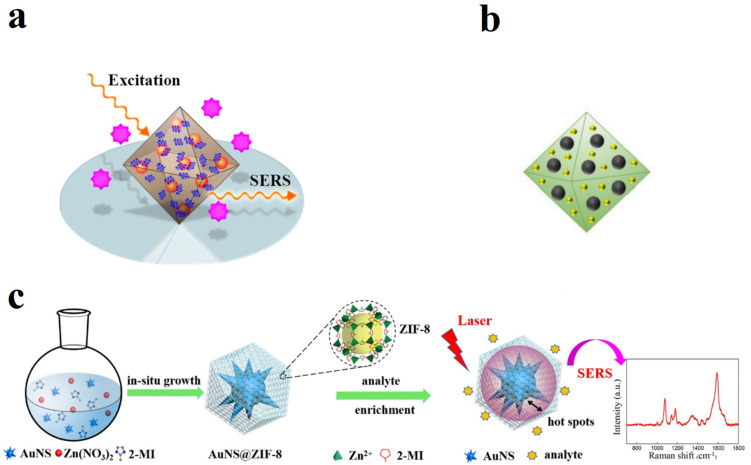
Water pollution detection and harmful gas detection in the environment (**a**) Au/MIL-101 (Cr) substrate [68]. (**b**) Au/Fe_3_O_4_/MIL-101 (Cr) substrate [46]. (**c**) AuNS@ZIF-8 Substrate preparation and SERS detection [50].

Hu et al. [68] encapsulated Au nanoparticles in the interior of MIL-101 (Cr) by solution impregnation and growth. The AuNPs/MIL-101 (Cr) substrate combines the local surface plasmon resonance characteristics of AuNP with the excellent adsorption capacity of MOF. By effectively pre-enriching the analyte near the electromagnetic field on the active metal surface of SERS, it becomes a highly sensitive SERS substrate (Figure 3a). Due to the protection of the MOF shell, the substrate also shows high stability and reproducibility. Through immersion of 4,4′-bipyridine and poly(4-vinylpyridine) in a solution of equal concentration and Raman spectroscopy analysis, it was observed that 4,4′-bipyridine had stronger Raman signals on the substrate, while poly(4-vinylpyridine) had negligible Raman signals. This suggests that the substrate can selectively enhance the Raman signals of small molecules, making them more detectable. Thanks to the synergistic effect of MIL-101 and embedded Au nanoparticles, the detection sensitivity of SERS is greatly improved, and the detection limit of phenylenediamine in a water environment is as low as 0.1 ng/mL. Based on the solution impregnation method, we reported super sensitive and high stability magnetic Au/Fe_3_O_4_/MIL-101 (Cr) (Figure 3b) composite SERS substrate for the detection of sulfapyridine in river water. The detection limit of the substrate for rhodamine 6G (R6G) is as low as 10^−8^ M, and the relative standard deviation is only 4.48%, demonstrating the substrate’s excellent SERS performance and consistency. When the concentration of sulfapyridine in river water is 10^−6^ M, its main characteristic peak can be detected at the Raman shift of 1001 cm^−1^ and 1078 cm^−1^. At the same time, the Raman spectrum of the river water was measured as a blank control, and it was found that there was no visible signal at the Raman shift of the characteristic peak. Therefore, the substrate provides the possibility for rapid and sensitive detection of sulfapyridine residues in the environment [46].

In addition, the MOF-based SERS substrate can not only quantitatively analyze the organic pollutants in the water environment but also detect the harmful and toxic gases in the environment with high sensitivity. MOF adsorbs gas molecules onto the composite interface of MOF and metal nanoparticles, which solves the problem of weak gas absorption of metal nanoparticles. Fu et al. [50] prepared a porous zeolite imidazolate framework (ZIF-8) shell by in situ growth on gold nanostars (AuNSs) to capture formaldehyde molecules (Figure 3c). Compared with the previous SERS detection methods, the layered porous structure of ZIF-8 core–shell can reduce the flow rate of formaldehyde molecules and change the direction of formaldehyde molecules, and prolong the contact time between formaldehyde molecules and matrix, which makes the analyte molecules easy to approach hot spots. With the help of the ZIF-8 shell, the AuNS@ZIF-8 substrate showed high sensitivity to formaldehyde, and the lowest detection level was almost 1 ppb. This provides some beneficial research for the detection of small gas molecules. Similarly, Lafuente et al. [69] used ZIF-8 for the successful capture of toxic gases. Their experiment involved slowing down the flow rate of gas on the surface of metal nanoparticles, which exhibited fast response time, low detection limit, excellent repeatability and recyclability in Surface-Enhanced Raman Scattering (SERS) response to sarin and mustard gas. The film produced from Au@Ag@ZIF-8 can detect as low as 0.2 parts per billion (ppb), making it an excellent tool for the sensitive detection of dimethyl methylphosphonate (DMMP) in the air. In addition, when we use MOF to capture the gas, the thickness of the MOF layer and the spacing of nanoparticles are also issues we need to consider. Koh et al. [70] designed a ZIF-encapsulated Ag nanocube array SERS substrate for nanoscale aggregation of gas molecules and EM fields in three-dimensional space. The hot spot area is enhanced by gas enrichment and controlling the plasma coupling between adjacent Ag nanocubes. Plasma nose with AG@ZIF can identify a range of volatile organic compounds (VOC) commonly emitted in industries, including chloroform and 2-naphthalene mercaptan, by analyzing their unique vibration characteristics. With detection limits reaching parts per million (ppm) levels, this technique offers a sensitive and efficient method for VOC detection. In addition to detection, real-time monitoring of gas molecules is also a very popular application. Phan Quang et al. [51] have demonstrated a real-time stand-off Surface-Enhanced Raman Scattering (SERS) spectroscopy for remote and multiplex detection of atmospheric airborne species by integrating a long-range optic system with a 3D analyte-sorbing metal–organic framework (MOF)-integrated SERS platform. This platform employs Ag@MOF core–shell nanoparticles that self-assemble into a 3D plasmonic architecture with a micrometer-thick SERS hotspot capable of actively sorbing and rapidly detecting aerosols, gas and volatile organic compounds down to parts-per-billion levels, even at a distance up to 10 m apart. The platform is highly sensitive to changes in atmospheric content, as demonstrated in the temporal monitoring of gaseous CO2 in several cycles.

### 3.2. Disease Diagnosis

Due to its rich fingerprints, resistance to photobleaching and photodegradation and non-invasiveness, SERS has great application prospects in the detection of biomolecules [71,72,73]. SERS is a trace detection technology extended to single molecule detection [74], which can provide fingerprint molecular information of biomarkers in real time and quickly for disease detection. The SERS substrate based on MOF can enrich, capture, distinguish and screen biomarkers in body fluid in vitro, such as exhaled breath, sweat and tears, so it can realize early diagnosis of many diseases [75].

**Figure 4:**
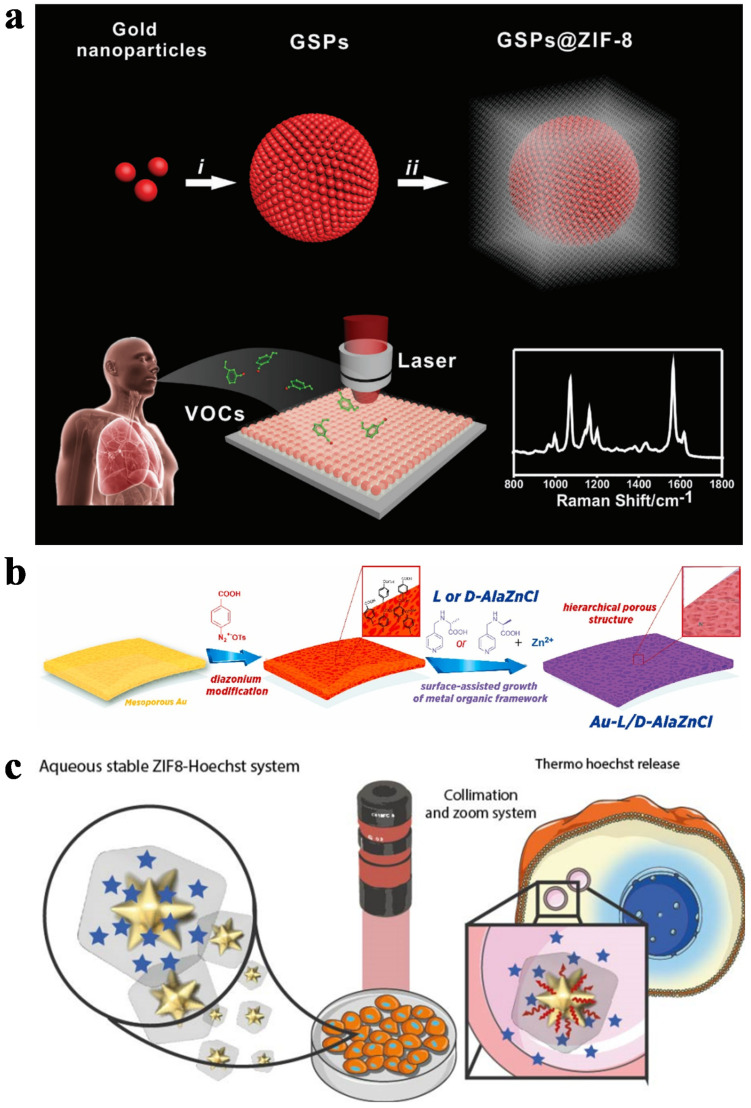
(**a**) GSPs@ZIF-8 SERS detection: (i) gold nanoparticles assembled into GSPs, (ii) ZIF-8 shell coated on GSP surface. [76]. (**b**) Schematic diagram of preparing layered porous AlaZnCl-Au film [77]. (**c**) Au@ZIF-8 Drug release and monitoring process [78].

In the face of the diagnosis of early lung cancer, Qiao et al. [76] prepared GSPs@ZIF-8. The substrate is used for the adsorption of gaseous aldehydes as a cancer-detection indicator(Figure 4a). The use of GSPS@ZIF-8 SERS substrate has enabled highly sensitive and selective detection of aldehydes with small Raman cross sections in the exhalation of lung cancer patients. GSPs@ZIF-8 exhibits additional Raman enhancement, which is attributed not only to the precise arrangement of nanoparticles but also to the change in electromagnetic field penetration depth that enhances the Raman signal of previously unresolved analytes. With the powerful adsorption capacity of ZIF-8, the flow rate of gas biomarkers is slowed down. However, the capture of gas depends on 4-aminothiophenol (4-ATP) grafted onto gold superparticle (GSP), so it can selectively detect the biomarker of lung cancer at the level of one billionth (ppb), which solves the problem that gas molecules are easily lost on the solid substrate. MOF-based SERS substrate can also have excellent chemical recognition and discrimination in the face of biological samples of biomolecules (such as serum or plasma components). Guselnikova et al. [77] prepared layered porous hybrid membranes by combining mesoporous plasma gold films and micropores with the chiral metal–organic framework (HMOF) (Figure 4b). Due to the double enhancement mechanism (physical enhancement of mesoporous Au nanostructures and chemical enhancement of HMOF), chemical recognition of HMOF and identification function of biological samples containing large biomolecules, such as blood components, the proposed layered porous substrate makes the detection value of pseudoephedrine in undiluted plasma very low (10^−12^ M).

In medical treatment, drug carriers based on MOF have also made great progress. Horcajada et al. [78] Au@ZIF-8 nano-composite materials are used to monitor the process of encapsulating active molecules in living cells and to drive the release of thermoplastic elements (Figure 4c). The encapsulated amphiphilic polymer has SERS imaging and photothermal effect, which can prevent ZIF-8 degradation and drug leakage into water medium or living cells. It has also been demonstrated that the molecular release mechanism relies on the absorption of plasma that is coupled to the core of the gold nanostar by using near-infrared light. This produces a local temperature gradient, leading to thermal diffusion of bis benzo imide. In addition, the MOF substrate of composite nanoparticles with a stable water phase can be used for multifunctional SERS imaging. For example, De et al. [79] packaged in ZIF-8 Au@AgNR and SERS recorder to form a SERS probe for in vitro imaging and various biological detection. The performance of the newly developed SERS tag was verified by the powerful capture of the ZIF-8 shell and the enhancement of the Raman signal of the core–shell structure, and the detection of the cell surface receptors EGFR and CD4 wake-up SERS.

### 3.3. Agriculture

Pesticides are often used in agricultural production to improve production efficiency. The typical detection methods of pesticides are based on enzyme-linked immunosorbent assay (ELISA) [80,81], HPLC or GC Mass Spectrometry [82,83], Surface Plasmon Resonance [84] and Fluorescence Spectroscopy [85]. None of these methods are suitable for on-site analysis. Surface-enhanced Raman spectroscopy has high sensitivity [52], reliable repeatability and selectivity in actual sample analysis [86]. Due to its unique porous structure and good dispersion, MOF can successfully adsorb pesticide molecules and enhance analytical signals [87].

**Figure 5:**
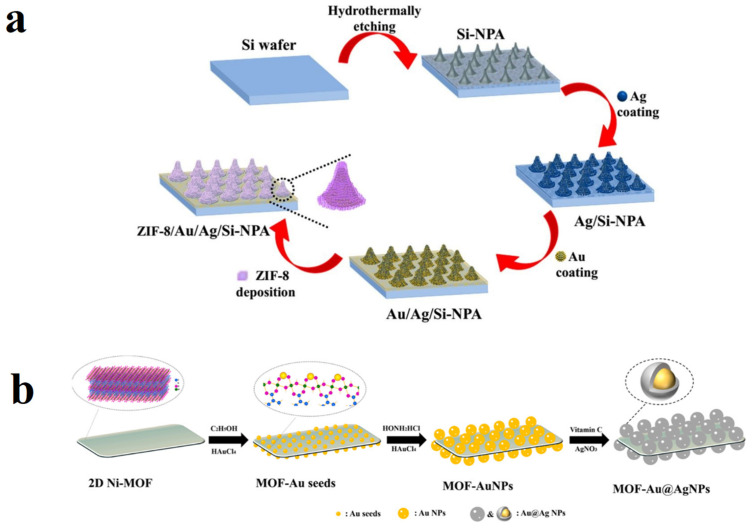
(**a**) Schematic diagram of preparation of ZIF-8/Au-Ag/Si-NPA active substrate [89]. (**b**) 2D Ni-MOF-Au@AgNPS Preparation of composite materials [55].

Cao et al. [88] synthesized three types of AuNP/MOF (MOF-199, UiO-66, UiO-67) composites to study the interaction between acetamiprid insecticides and bridging molecules of MOF. In this case, acetamiprid is used to evaluate the properties of SERS substrates. The LOD of the three composites are 0.02 μM, 0.009 μM and 0.02 μM, respectively, which can meet the detection requirements of acetamiprid. Pentachlorophenol (PCP) is a commonly used insecticide, which has a great application in wood preservatives, but it is easy to cause poisoning if people inhale it for a long time. Based on the detection of pentachlorophenol, Yan et al. [89] grew ZIF-8 films on Au-Ag/Si-NPA (Figure 5a) substrates by layer-by-layer growth method, and successfully prepared ZIF-8/Au-Ag/Si-NPASERS substrates. It can effectively capture a small amount of PCP molecules close to hot spots, thus amplifying the SERS signal, and the detection limit of SERS is as low as 10^−13^ M. Moreover, the substrate showed good uniformity with a relative standard deviation (RSD) of 8.7% and good selectivity. PCP detection is hardly interfered with by the coexisting organic compounds. The high SERS performance may be due to the enrichment effect of the ZIF-8 film. The ZIF-8 film could capture and enrich the trace PCP molecules by electrostatic interaction between the negatively charged PCP and the positively charged ZIF-8.

After the growth of crops, the remaining pesticides in the soil are also a big problem. Lai et al. [55] developed a method for in situ growth of core–shell Au@Ag nanoparticles on two-dimensional Ni-MOF nano-thin films (Ni-MOF-Au@AgNPs) (Figure 5b) as high-performance SERS substrates. It has good sensitivity, stability and charge selectivity. The Ni-MOF-Au@AgNPs SERS substrate displayed a dense distribution of Au@AgNPs, leading to significantly high enhancement factors of 2.2 × 10^6^, 3.7 × 10^5^, and 9.5 × 10^5^ for thiram, diquat and paraquat, respectively. This enabled the Ni-MOF-Au@AgNPs substrate to accurately quantify these compounds in the concentration range of 0.01 to 50 mg/L, with exceptionally low detection limits of 87.1, 188.8 and 8.9 μg/L, correspondingly. Food sample analysis using this substrate yielded recoveries of thiram, diquat and paraquat ranging from 80.6% to 105%, 87.8% to 102% and 80.0% to 113%, with relative standard deviations of 1.0% to 6.2%, 1.4% to 7.7% and 1.6% to 7.4% (n = 3) for each compound. Based on the core and shell structure of MOF, gas can be enriched and volatile pesticides can be effectively detected. Zhou et al. [90] prepared a sea urchin-like Au–Ag particle wrapped in ZIF-8 (UAANs@ZIF-8). The SERS effect, which is significantly enhanced by ZIF-8 encapsulation and the enrichment effect of its shell, exhibits a power function increase with decreasing HCH concentration, particularly below 10^−6^ M. The ever-increasing enrichment effect of ZIF-8 to HCH molecules explains this phenomenon. This matrix has a detection limit of less than 1.5 ppb for trace volatile HCH pesticides in SERS detection.

### 3.4. Food Safety

**Figure 6:**
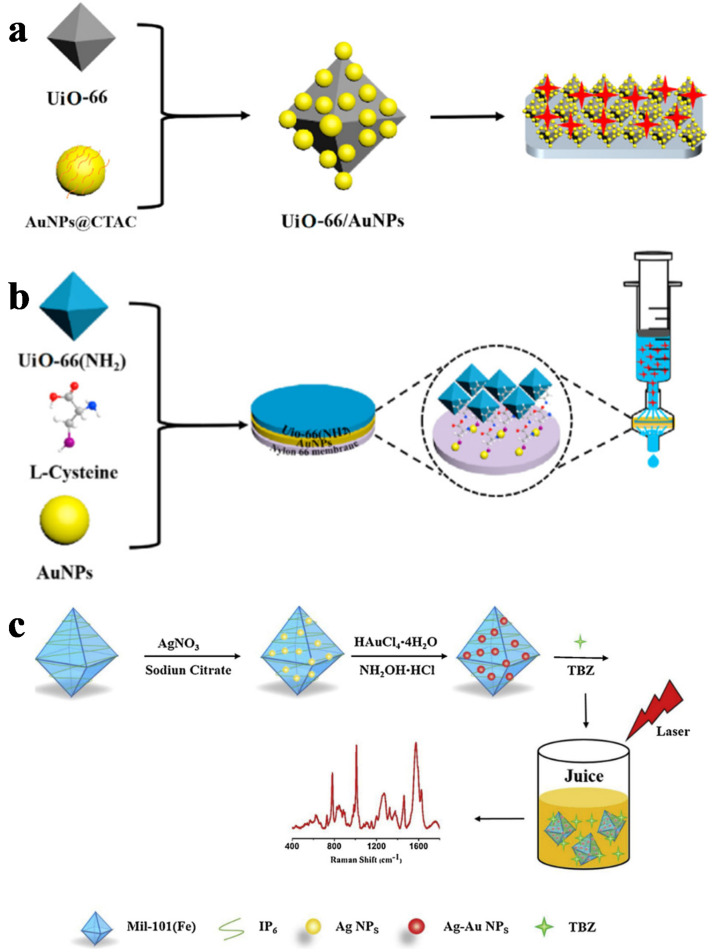
(**a**) UiO-66/AuNPs [96] (**b**) UiO-66 (NH_2_)/AuNPs/Nylon-66 [96] (**c**) Preparation of Ag-Au-IP_6_-MIL-101 (Fe) and SERS determination of TBZ in fruit juice [97].

The increasingly serious food safety problems have attracted more and more attention, which is a serious threat to human health. The non-selective enhancement of traditional precious metal substrates and the complexity of samples seriously hinder the practical application of SERS in food safety analysis [91]. Surface-enhanced Raman scattering (SERS) technology has attracted much attention in the field of food safety due to its simple operation [92], high sensitivity [93] and in situ and non-invasive detection [94]. Because MOF has a large surface area, adjustable aperture and easily adjustable structure, its interaction with the target can provide more unsaturated sites, which is conducive to SERS signal amplification [95]. Therefore, there is an urgent need to eliminate interference and improve the accuracy of SERS determination based on developing efficient sample preparation technology. For example, heterocyclic amines containing 2-amino-3-dimethyl-3-imidazolquinoline (MeIQ) in barbecue meats are carcinogens and are usually produced during barbecue. In the face of such a large number of complex samples, Fu et al. [96] developed a UiO-66/AuNPs suspension matrix (Figure 6a) and UiO-66 (NH_2_)/AuNPs/Nylon-66 (Figure 6b) flexible membrane matrix for practical SERS analysis of food samples. The preparation of the UiO-66/AuNPs suspension matrix depends on electrostatic interaction. The substrate of UiO-66 (NH_2_)/AuNPs/Nylon-6 flexible membrane was assembled by UiO-66 (NH_2_) MOF and AuNPs in the presence of cysteine. The established method was applied to the actual determination of MeIQ in three barbecue samples. The corresponding concentrations of trace MeIQ in two positive samples were 2.07 and 2.21 mg/kg, respectively. Moreover, the residual antibiotics in animal meat are also a problem that needs to be paid attention to. We prepared Ag@MIL-101 (Cr) thin film SERS substrate. [47]. An Ag/MIL-101 (Cr) thin film substrate exhibiting SERS activity was synthesized via the reduction of silver ions to silver nanoparticles on a MIL-101 (Cr) thin film under UV irradiation. This substrate was capable of detecting the Raman signal of 4-ATP at a concentration as low as 10^−11^ mol/L, with RSD values of 5.03% and 4.66% at Raman shifts of 1141 cm^−1^ and 1434 cm^−1^, respectively. Moreover, the substrate was utilized for the SERS detection of nitrofurantoin and exhibited a minimum detection limit of 10^−11^ mol/L.

Similarly, Xu et al. [97] prepared Ag-Au-IP_6_-MIL-101 (Fe) (Figure 6c) in situ on the MOF surface. Some target molecules containing N or S atoms can approach the surface of Au-IP6-MIL-101 (Fe) substrate through strong interaction with Fe^3+^, thus improving the detection sensitivity of SERS. The rapid detection of thiabendazole in fruit juice has a good linearity in the range of 1.5 ppm to 75 ppm and the correlation coefficient (R2) is 0.986, and the detection limit of TBZ in fruit juice samples reaches 50 ppb, meeting the requirements of national standards. In addition to the residual antibiotics in meat, stains in food may pose a threat to human health. Wu et al. [35] prepared UiO-66 (NH_2_)@Au multifunctional SERS substrate (Figure 6c) for quantitative detection of edible carmine (NC) and methyl orange (OII) using UiO-66 (NH_2_)@Au SERS substrate. These results show that the integration of MOF–SERS detection will effectively improve the precision and accuracy of analysis and promote the application of MOF–SERS in food safety analysis.

### 3.5. Biosensors

**Figure 7:**
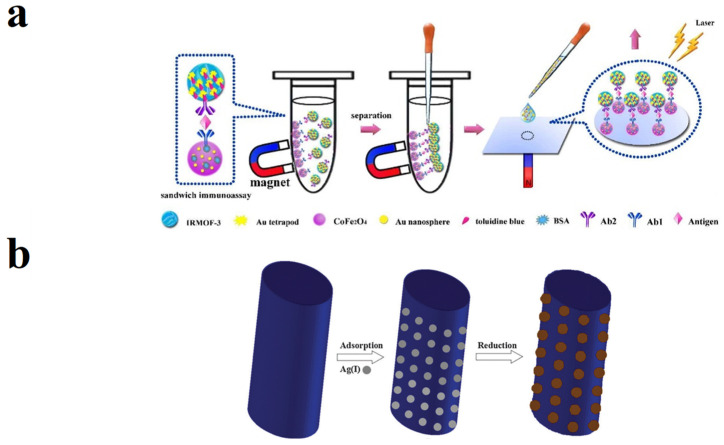
(**a**) Schematic diagram of SERS-based immunosensor for detecting NT-proBNP [98], (**b**) Schematic diagram of h-Ag NP formation on MOF [100].

SERS substrates based on MOF can be used as SERS-based immunosensors for the selective detection of specific biomarkers. He et al. [98] CoFe_2_O_4_@AuNPS@Ab1 magnetic substrate is a capture probe, IRMOF-3@ AuTPs@ TB@Ab2 The complex is a SERS immunosensor with SERE tag, which was used to detect NT-proBNP (Figure 7a). Zhang et al. designed a SERS immunosensor to detect NT-proBNP, CoFe_2_O_4_@AuNPS@Ab1. The magnetic substrate is a capture probe, IRMOF-3@AuTPs@TB@Ab2. The compound is a SERE label (Figure 7a). The detection limit of the sensor was 0.75 fg mL-1, with six orders of magnitude in the range of 1 fg mL^−1^. IRMOF-3@AuTPS @TB has a large specific surface area of IRMOF-3 and a large number of hot spots generated by Au TPS, which improves the sensitivity of SERS immunoassay. In addition, Wu et al. [99] constructed a DNA-functionalized MOF proportional SERS sensor based on stimulation response for the detection of adenosine triphosphate (ATP). The detection range of this method for ATP is 1 × 10^−9^~2 × 10^−7^ M, and the detection limit is 4 × 10^−10^ M. Compared to the typical SERS detection based on a single signal response, using a ratio SERS biological sensor strategy provides a lower detection limit and superior performance, which may have the potential to be applied in the detection of other biological molecules or metal ions. In addition, special metal particles loaded on MOF can also be used for the biological detection of SERS. Kuang et al. [100] stabilized h-AgNPS to MOF material for the first time as a new SERS sensor (Figure 7b), and the chiral arrangement of silver nanoparticles loaded on MOF-1 carrier enables them to have enantioselective interaction with D/L-cysteine and D/L-asparagine, forming complexes with significantly different SERS effects. The enhancement of the SERS intensities is attributed to the matching of the stereoscopic configuration between L-Cys and the h-AgNPs. The results show that the material can be used as a new SERS sensor for enantioselective recognition. SERS biosensors based on MOF can also be used for gas detection. Fu et al. [101] believe that MIL-100 (Fe) is a special SERS matrix used for detecting volatile organic compounds (VOCs). The substrate has a detection limit (LOD) of 2.5 ppm for toluene, and the addition of Au nanoparticles to the substrate helps reduce the “hot spot” to 0.48 ppb, resulting in an impressive enhancement factor of 10^10^. The researchers also discovered that MIL-100 (Fe) has distinct “sensor array” properties, allowing for the detection of multiple VOCs. Furthermore, the substrate can be easily modified and scaled through the use of foreign metal elements.

## 4. Conclusions and Prospect

In recent years, SERS substrates based on MOF have been effectively applied in various fields. In this review, we introduced the unique role of MOF in SERS, the classification of SERS substrates based on MOF, and the application of MOF–SERS substrates in the environment, biomedicine, agriculture, etc. The introduction of MOF into the SERS substrate can realize the synergistic effect of electromagnetic effect (EM) and chemical effect (CM). Compared with other assays, it has many unique advantages, such as efficient molecular capture, improved stability, improved sensitivity and convenience (Table 2). Therefore, the SERS substrate based on MOF can obtain more accurate results in the face of complex environments. The potential development directions for MOF–SERS in the future are as follows: There is relatively little research on the application of a single MOF to SERS, and the research on the mechanism is not thorough. This is a direction worthy of further research; Application of MOF–SERS substrates in the field of photocatalysis: MOF–SERS substrates have high structural controllability and surface modification, and can be used to construct functional photocatalytic materials, achieving high selectivity and efficient degradation of specific organic compounds; Application of MOF–SERS substrate in the field of nanoelectronics: As a very sensitive probe, MOF–SERS substrate can be used for high sensitivity and precision nanoelectronics measurements. Future research can explore the widespread application of MOF–SERS substrates in the field of nanoelectronics.

Although the SERS substrate based on MOF has great potential, it also faces inevitable problems. First, there are few studies on the application of a single MOF in SERS detection, which makes us lack understanding of the functions of different MOF materials in SERS, and the Raman characteristic peaks of MOF itself also have an impact on the detection results. These signals will interfere with the Raman signal, thus limiting their use in complex sample detection. Secondly, MOF composites with excellent functions are also what we have been looking for. MOF is hybridized with polymer to combine the flexibility, thermal stability, chemical stability and photoelectric performance of the polymer. It is combined with graphene, nanotubes and other materials to improve conductivity. Third, the MOF–SERS substrate used for SERS detection is difficult to recover and reuse, which makes the cost high. Fourth, in terms of data processing, artificial intelligence and principal component analysis, they can effectively help us process and extract SERS signals. However, we need to think more about how to increase the recognition sites of biological macromolecules and establish a unified standard data processing method. Although there are some problems at present, the MOF–SERS platform presents a bright and very promising prospect. With the development of more materials and methods, we believe that MOF can be better applied to the SERS field.

## Figures and Tables

**Table 1:** Research progress on different types of MOF-SERS.

Type	Year	Substrates	Target	EF/LOD	Ref.
MOFs	2013	MIL-100 (Al)	MO	-	[36]
	2018	ZIF-67	R6G	10^−8^ M	[32]
	2021	Mo-MOF	Crystal Violet	1.33 × 10^5^	[59]
MOFs/Semiconductor	2021	GO/MIL-101 (Fe)	-	-	[60]
MOF surface induced precious metal particles	2020	UiO-66 (NH_2_)@Au	Orange II	0.0546 mg/L	[35]
	2021	UIO-66@Ag NPs	Di-(2-ethylhexyl) phthalate	3 × 10^−12^ M	[61]
	2022	Au/Fe_3_O_4_/MIL-101 (Cr)	Sulfapyridine	10^−6^ M	[46]
Surface induced MOF shell of metal nanoparticles	2020	Ag@Ni-MOF-1	Cysteine	-	[53]
	2021	Au@ZIF-8	Toluene	-	[48]
	2022	Au nanorod@Zr-MOF	4′-mercaptobiphenylcarbonitrile	2 × 10^−10^ M	[62]
Self assembly of MOF and precious metals	2019	Au/MOF-74	4-nitrothiophenol	69 nM	[58]
	2021	AgNPs/MIL-101 (Fe)	Paraquat	2.09 × 10^9^ M	[57]

**Table 2:** Comparison between MOFS and other SERS substrates.

Material	Detector	EF	Stability	Selectivity	LOD (M)	Ref.
AuNPs	Procaine	-	Stable	Size, structure	10^−10^ M	[102]
AgNPs	Oxytetracycline	-	Not stable (Easy to oxidize)	Size, structure	5 ppb	[103]
Cu	Crystal violet	3.9 × 10^3^	Not stable (Easy to oxidize)	Size, structure	10^–8^ M	[104]
Graphene	Rhodamine B	-	Stable	Charge, structure	10^−11^ M	[105]
MoS_2_	4-mercaptopyridine	>3 × 10^5^	Stable	-	-	[106]
MOFs	Paraquat	2.09 × 10^9^	Stable	Size, structure, charge	10^−12^ M	[57]

## Data Availability

Not applicable.
